# Cyclists injured in traffic crashes in Hong Kong: A call for action

**DOI:** 10.1371/journal.pone.0220785

**Published:** 2019-08-09

**Authors:** Pengpeng Xu, Ni Dong, S. C. Wong, Helai Huang

**Affiliations:** 1 Department of Civil Engineering, The University of Hong Kong, Hong Kong, China; 2 School of Transportation and Logistics, Southwest Jiaotong University, Chengdu, Sichuan, China; 3 School of Traffic and Transportation Engineering, Central South University, Changsha, Hunan, China; University of British Columbia, CANADA

## Abstract

**Background:**

Perceived as a minor transportation mode mainly for recreation, cycling and its related safety issues have not been treated as a citywide concern in Hong Kong and have thus received inadequate research efforts. Our study aimed to illuminate the safety challenges faced by cyclists in Hong Kong.

**Methods:**

We examined the police crash records from 1998 to 2017 and developed a Bayesian Poisson state space model to evaluate the longitudinal change in traffic injuries to cyclists. We then used quasi-induced exposure to measure the annual relative risk of crash involvement for cycling. Based on an officially published travel characteristics survey, we further measured the risk of injury for cycling per minutes cycled.

**Results:**

Between 1998 and 2017, Hong Kong witnessed a more than twofold increase in the number of cyclist injuries, with an average annual increase rate of 5.18% (95% CI: 0.53%–12.77%). By 2017, cyclists were 2.21 (1.82–2.69) times more likely to be involved in traffic crashes than in 1998. Per 10 million minutes, the injury rates for cycling were 28.64 (27.43–29.70) and 42.54 (41.07–44.02) on weekdays during 2001–2003 and 2010–2012. After adjusting for sex and age groups, cyclists were 1.95 (1.43–2.61) times more likely to be injured in 2010–2012 than in 2001–2003. Per minutes traveled, cyclists also sustained significantly higher risks of fatality and injury than pedestrians, private car drivers and passengers, taxi passengers, public bus passengers, and minibus passengers. A comparison of Hong Kong with other regions suggests that Hong Kong is among the most dangerous areas for cycling in terms of fatality rate per minutes cycled.

**Conclusions:**

Cyclist injuries have become a substantial public health burden in Hong Kong. A range of countermeasures with proven effectiveness should be promptly implemented to improve the safety of these vulnerable road users.

## Introduction

Among the active modes of transportation, cycling has the advantages of reducing traffic congestion, greenhouse gas emissions, and traffic noise, fostering a more livable community [1–2]. Around the world, cycling is also a popular physical and recreational activity, particularly among children and adolescents. With the increasing number of short-distance trips, growing levels of traffic congestion, and higher parking costs in metropolitan areas, people are being increasingly encouraged to cycle more as a viable and affordable mode of transportation [3].

Despite the documented benefits of cycling, Hong Kong, as a highly urbanized area with a well-established public transit system, has seen a relatively low bicycle use compared with other metropolitan areas. A recent official survey of travel characteristics estimated that cycling accounted for less than 1% of all trips made by local residents on normal weekdays [4]. Perceived as a minor transportation mode mainly for recreation, cycling and its related safety issues have not yet been treated as a citywide concern and have thus received inadequate research efforts [5–8].

However, the narrative changes if we consider the recent trend in traffic injuries suffered by cyclists. According to police reports [9], the number of cyclists injured in crashes has more than doubled in the last two decades, increasing from 683 in 1998 to 1,779 in 2017. Its share of traffic-related casualties rose accordingly from 3.58% to 8.95%. By comparison, during the same period, the number of pedestrian casualties dropped by roughly 37% from 4,932 to 3,090. This safety disparity is especially alarming given that the mode share of walking was almost 23 times that of cycling [4].

This study therefore aims to illuminate the safety challenges faced by cyclists in Hong Kong. By examining police crash records from 1998 to 2017, we develop a Bayesian Poisson state space model to evaluate the longitudinal change in traffic injuries to cyclists over the past two decades. We then use quasi-induced exposure to quantify the relative risk of crash involvement for cycling annually during the period of interest. Based on the travel characteristics survey, we also estimate the fatal injury rate for cyclists per billion minutes and their injury rate per 10 million minutes on normal weekdays during 2001–2003 and 2010–2012. A deeper understanding of the recent changes in the safety burden sustained by cyclists will undoubtedly stimulate the formulation and implementation of evidence-based safety strategies for these vulnerable road users.

## Methods

We obtained crash data from the Traffic Road Accident Database, which is maintained by the Hong Kong Police Force and the Hong Kong Transport Department [10]. These data are routinely collected by the police at the scenes of crashes. Only crashes resulting in injuries on public roads are recorded in the database. Casualties who died immediately at the scene or within 30 days of the collision are counted as fatalities.

This study was approved by The Human Research Ethics Committee for Non-Clinical Faculties, The University of Hong Kong.

We developed a Bayesian Poisson state space model [11] to assess the longitudinal change in traffic injuries to cyclists over the past two decades. Let *Y*_*t*_ denote the number of cyclists injured in traffic crashes in the *t*th(*t* = 1,2,…,20) year. Given the random, non-negative, and integral nature of injury counts, we have:

$$\begin{array}{l} Y_{t}∼\mathrm{Poisson}(\lambda_{t})\\ \log(\lambda_{t})=\alpha_{t}+\beta \times t \end{array}$$ (1)

where λ_*t*_ refers to the parameter of the Poisson distribution, i.e., the expected number of cyclists injured. We model the time effects deterministically as a fixed linear trend with coefficient *β* [12]. As a result, the average annual increase rate can be estimated as $100\times (e^{\hat{\beta }}-1)\%$.

We handle the potential temporal correlation by allowing the intercept term to vary over time [13]:

$$\begin{array}{l} \alpha_{t+1}=\alpha_{t}+s_{t}\\ s_{t}∼\mathrm{Normal}(0,\sigma^{2})\\ \alpha_{1}\sim \mathrm{Normal}(0,0.0001) \end{array}$$ (2)

To obtain the full Bayesian posterior estimates, the prior distributions are required to be specified. Due to the absence of sufficient prior knowledge, a non-informative prior, i.e., normal distribution (0,0.0001), was specified for *β* [14,15]. The variance parameter *σ* was assigned as a uniform distribution (0,100) [16–18].

For comparison, we also estimated the same model with the number of pedestrians, motorcyclists, private car drivers, private car passengers, taxi passengers, public bus passengers, and minibus passengers injured in traffic crashes as the dependent variable, respectively.

The freeware software OpenBUGS (version 3.2.3) [19] was used to calibrate the models. Two parallel chains with diverse starting points were tracked. The first 5,000 iterations were discarded as burn-ins, and then 5,000 iterations were performed for each chain, resulting in a sample of distribution of 10,000 for each parameter. The model convergence was monitored by the Brooks-Gelman-Rubin statistic, visual examination of the Markov Chain Monte Carlo chains, and the ratios of Monte Carlo errors relative to the respective standard deviations of the estimates. As a rule of thumb, these ratios should be less than 0.05.

The police judge all individuals involved to be either responsible or not responsible for the collision. Therefore, to quantify the danger of cycling over the studied period, by assuming the non-responsible individuals to be a representative sample of the underlying population on the roads [20–22], we estimated the annual relative risk of crash involvement for cycling as:

$$RR_{cyclists}^{tvs.1998}=\frac{R_{cyclists}^{t}}{R_{cyclists}^{1998}}/\frac{N_{cyclists}^{t}}{N_{cyclists}^{1998}}$$ (3)

where $R_{cyclists}^{t}$ and $N_{cyclists}^{t}$ are the numbers of cyclists who were responsible or not responsible for the crashes in the *t*th(*t* = 2,3,…,20) year, respectively. Here, cyclists involved in crashes in the year 1998 serve as the baseline, and the corresponding numbers of cyclists who were responsible or not responsible are denoted as $R_{cyclists}^{1998}$ and $N_{cyclists}^{1998}$, respectively. The SAS software (version 9.4) was used to compute the unadjusted odds ratio (OR) and 95% confidence intervals (CI).

In addition to the quasi-induced exposure, we further measured the risk of injury for cycling as the fatal injury rate per billion minutes [23–25] and injury rate per 10 million minutes [23,26–28]. The travel characteristics survey released by the Hong Kong Transport Department provides a reliable and straightforward means to estimate the exposure for various road users [8,29,30]. In the latest survey, a sample of 35,401 households (1.5% of the population) was randomly enumerated between September 2011 and January 2012 [4]. Respondents aged 2 or above were asked to recall all types of activities they engaged in on the preceding weekday (excluding Saturdays, Sundays, and public holidays). General trip information, including trip duration and associated travel mode, was recorded accordingly. The collected trip records were then extrapolated to the entire population and were further adjusted for underreporting by comparison with independent transportation statistics [31]. A similar survey was conducted between September and December 2002 with a total of 30,005 households (1.4% of the population) successfully enumerated [32]. We therefore estimated the risk of injury for cycling on normal weekdays by dividing the reported number of cyclists injured or fatally injured in crashes during this period by the amount of time people spent cycling, and compared this risk with those of walking, riding a motorcycle, driving or taking a private car, taking a taxi, taking a public bus, and taking a minibus. We also compared the rate of fatal injury for cycling in Hong Kong with that in other regions worldwide [25,33,34]. Following Tin Tin et al. [26], Mindell et al. [27], and Santamariña-Rubio et al. [28], we estimated the 95% CI for these rates using the Poisson distribution with the SAS software (version 9.4).

## Results

**Fig 1:**
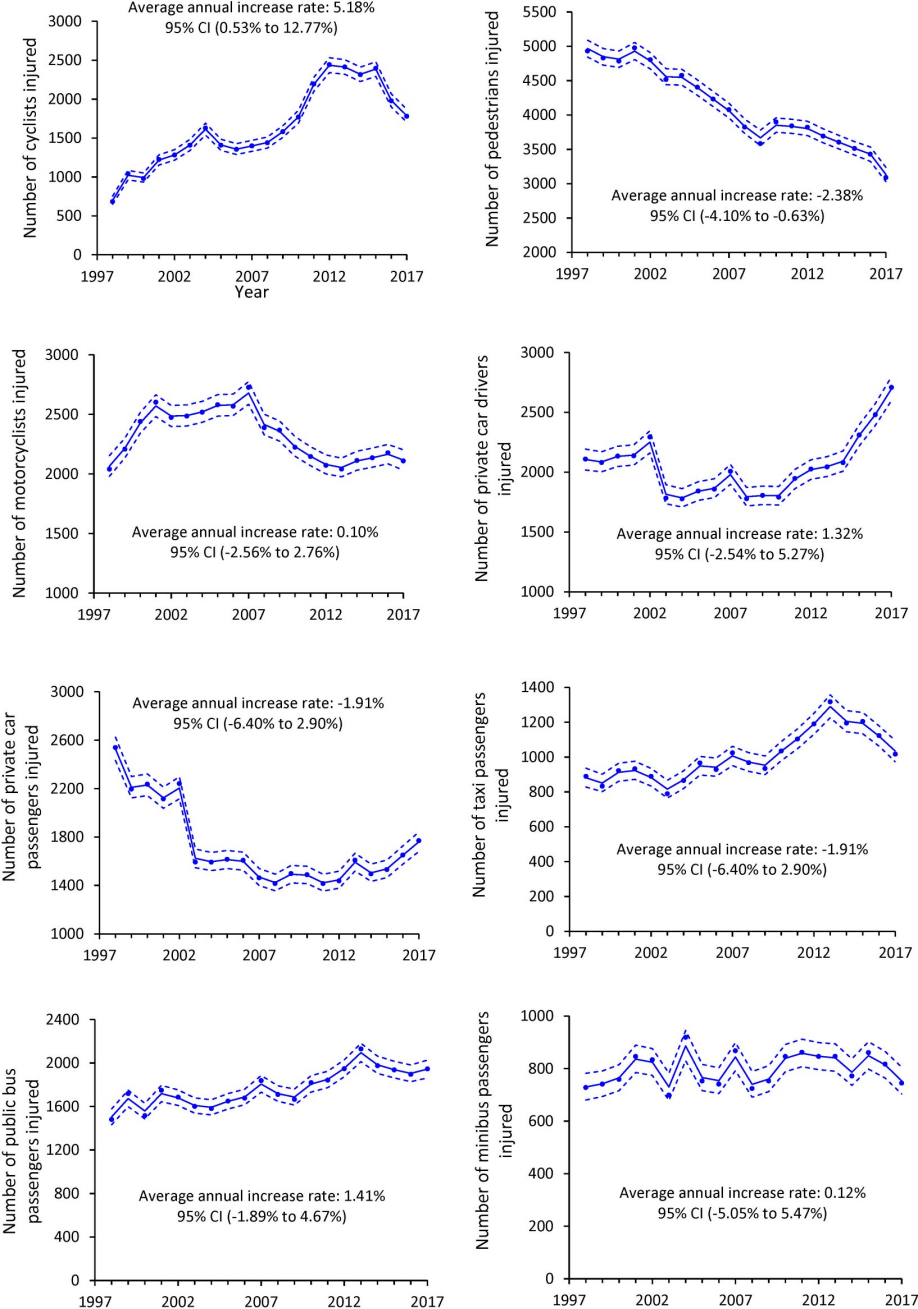
Observed and expected number of cyclists, pedestrians, motorcyclists, private car drivers, private car passengers, taxi passengers, public bus passengers, and minibus passengers injured in road traffic crashes, Hong Kong, 1998–2017 (dots: observed data; solid lines: expected data; dashed lines: 95% CI).

During 1998–2017, 32,709 cyclists were reported to be injured in traffic crashes, accounting for 8.36% of total traffic casualties in Hong Kong (see S1 Table). The number of cyclist injuries rose steadily from 683 to 1,779, with an average annual increase rate of 5.18% (95% CI: 0.53% to 12.77%) (see Fig 1). In contrast, a statistically significant declining trend was observed for pedestrian injuries (average annual increase rate: –2.38%; 95% CI: –4.10% to –0.63%), while the trends for injuries to motorcyclists, private car drivers, private car passengers, taxi passengers, public bus passengers, and minibus passengers remained stable.

**Fig 2:**
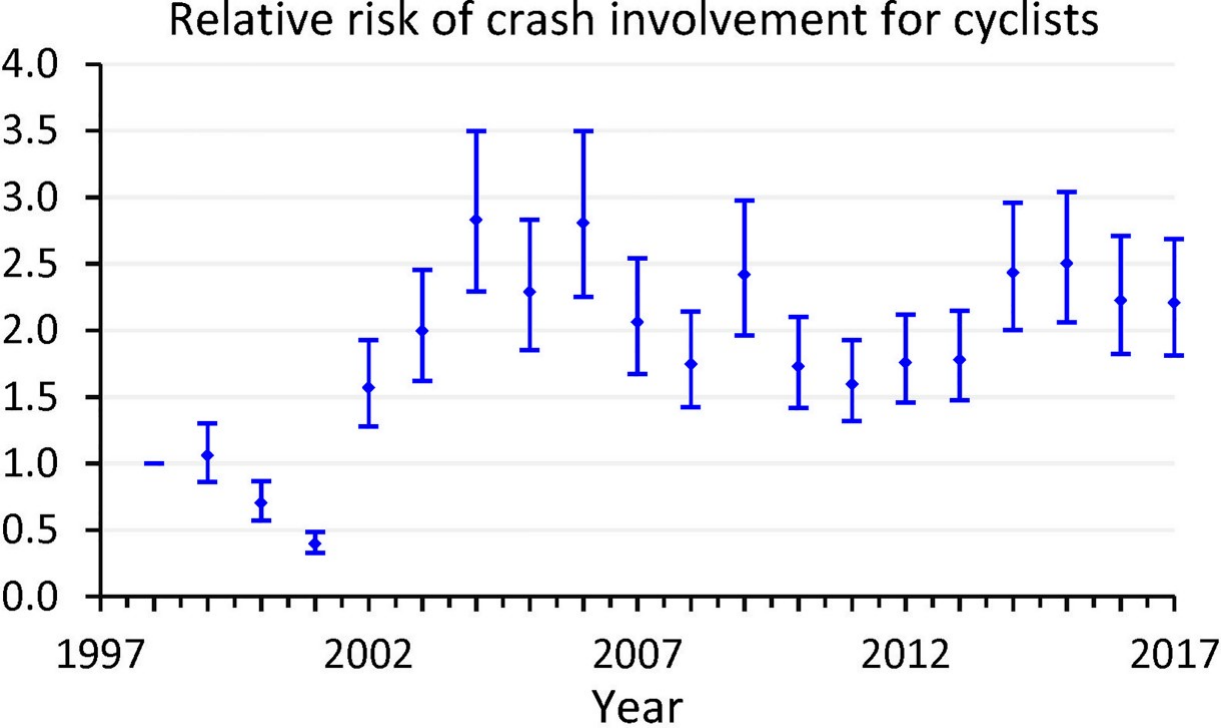
Risk of crash involvement for cyclists, measured by quasi-induced exposure, Hong Kong, 1998–2017 (dots: unadjusted OR; solid lines: 95% CI).

Measured by the quasi-induced exposure, the risk of crash involvement for cyclists decreased during 2000–2001 and increased substantially afterwards (see Fig 2 and S2 Table). By 2017, cyclists were 2.21 (95% CI: 1.82 to 2.69) times more likely to be involved in traffic crashes than in 1998.

As shown in Table 1, the fatal injury rate for cyclists was 27.94 (95% CI: 17.27 to 41.02) per billion minutes on normal weekdays during 2001–2003. This figure increased slightly to 33.96 (95% CI: 22.29 to 48.07) during 2010–2012. Likewise, the injury rates for cyclists were 28.64 (95% CI: 27.43 to 29.70) and 42.54 (95% CI: 41.07 to 44.02) per 10 million minutes on normal weekdays during 2001–2003 and 2010–2012, respectively. After adjusting for sex and age groups [24], cyclists were 1.95 (95% CI: 1.43 to 2.61) times more likely to be injured in 2010–2012 than in 2001–2003.

**Table 1:** A comparison of fatality and injury rates for cyclists on normal weekdays between 2001–2003 and 2010–2012.

	Fatality rate per billion minutes				Injury rate per 10 million minutes			
	Rate	95% CI	Ratio^†^	95% CI	Rate	95% CI	Ratio^†^	95% CI
2001−2003	27.94	(17.27, 41.02)	1.00	———	28.64	(27.43, 29.70)	1.00	———
2010−2012	33.96	(22.29, 48.07)	1.29	(0.26, 3.93)	42.54	(41.07, 44.02)	1.95	(1.43, 2.61)

^†^ Rate ratio adjusted for sex and age. Age is grouped as <17, 18–24, 24–34, 35–44, 45–54, 55–64, and >64.

As shown in Table 2, the rates of fatality and injury for cyclists vary sharply between urban and rural areas. Per billion minutes cycled, the fatality rates for cycling on normal weekdays during 2010–2012 were 223.80 (95% CI: 91.00 to 416.40) and 26.79 (15.94, 40.24) in urban and rural areas, respectively. The corresponding injury rates were 83.39 (95% CI: 73.80 to 93.85) and 40.15 (95% CI: 38.70 to 41.63) per 10 million minutes. After adjusting for sex and age groups [24], cycling in urban areas was 8.82 (95% CI: 3.14 to 18.67) and 2.08 (95% CI: 1.83 to 2.35) times more likely to result in fatal injury or injury than in rural areas.

**Table 2:** Fatality and injury rates for cyclists by areas on normal weekdays, Hong Kong, 2010–2012.

	Cycling time		Fatality rate per billion minutes				Injury rate per 10 million minutes			
	N^†^	%	Rate	95% CI	Ratio^‡^	95% CI	Rate	95% CI	Ratio^‡^	95% CI
Urban areas*	42,395	4.21	223.80	(91.00, 416.40)	8.82	(3.14, 18.67)	83.39	(73.80, 93.85)	2.08	(1.83, 2.35)
Rural areas	964,657	95.79	26.79	(15.94, 40.24)	1.00	———	40.15	(38.70, 41.63)	1.00	———

* Hong Kong Island and Kowloon are traditionally considered as urban areas, while the New Territories is regarded as a rural area.

^†^ Unit: minutes per weekday.

^‡^ Rate ratio adjusted for sex and age. Age is grouped as <17, 18–24, 24–34, 35–44, 45–54, 55–64, and >64.

A comparison of cyclists with other major road users indicated that cyclists sustained approximately 5.41 (95% CI: 2.33 to 15.23), 1.89 (95% CI: 0.65 to 8.87), 75.08 (95% CI: 27.38 to 344.59), 98.43 (95% CI: 30.81 to 717.36), 72.99 (95% CI: 25.23 to 380.23), 1269.20 (95% CI: 398.72 to 10790.98), and 492.13 (95% CI: 138.70 to 6441.15) times higher risks of fatality than pedestrians, motorcyclists, private car drivers, private car passengers, taxi passengers, public bus passengers, and minibus passengers per minutes traveled on normal weekdays during 2010–2012 (see Table 3). Similar but less pronounced results can be observed for injury rate. Per 10 million minutes traveled, the injury rate for cyclists was only surpassed by that for motorcyclists. After adjusting for sex and age groups [24], cyclists were overall 15.30 (95% CI: 9.17 to 29.38), 24.63 (95% CI: 14.09 to 49.26), 28.20 (95% CI: 16.64 to 53.97), 36.36 (95% CI: 21.44 to 67.75), 164.69 (95% CI: 99.01 to 298.15), and 88.81 (95% CI: 52.47 and 165.70) times more likely to be injured in traffic crashes than pedestrians, private car drivers, private car passengers, taxi passengers, public bus passengers, and minibus passengers on normal weekdays during 2010–2012.

**Table 3:** Fatality and injury rates for cyclists, pedestrians, motorcyclists, private car drivers, private car passengers, taxi passengers, public bus passengers, and minibus passengers on normal weekdays, Hong Kong, 2010–2012.

	Fatality rate per billion minutes				Injury rate per 10 million minutes			
	Rate	95% CI	Ratio^†^	95% CI	Rate	95% CI	Ratio^†^	95% CI
Cyclists	33.96	(22.29, 48.07)	1.00	———	42.54	(41.07, 44.02)	1.00	———
Pedestrians	6.18	(5.03, 7.45)	0.18	(0.07, 0.43)	3.32	(3.24, 3.41)	0.07	(0.03, 0.11)
Motorcyclists	19.68	(11.37, 30.10)	0.53	(0.11, 1.55)	50.75	(49.25, 52.29)	1.19	(0.53, 2.31)
Private car drivers	0.50	(0.25, 0.84)	0.013	(0.003, 0.037)	1.82	(1.76, 1.88)	0.04	(0.02, 0.07)
Private car passengers	0.30	(0.10, 0.60)	0.010	(0.001, 0.032)	2.44	(2.34, 2.54)	0.04	(0.02, 0.06)
Taxi passengers	0.48	(0.19, 0.90)	0.014	(0.003, 0.040)	1.67	(1.60, 1.74)	0.03	(0.01, 0.05)
Public bus passengers	0.03	(0.01, 0.07)	0.0008	(0.0001, 0.0025)	0.38	(0.37, 0.40)	0.006	(0.003, 0.0010)
Minibus passengers	0.06	(0.01, 0.15)	0.002	(0.0002, 0.0072)	0.60	(0.57, 0.63)	0.011	(0.006, 0.019)

^†^ Rate ratio adjusted for sex and age. Age is grouped as <17, 18–24, 24–34, 35–44, 45–54, 55–64, and >64.

**Fig 3:**
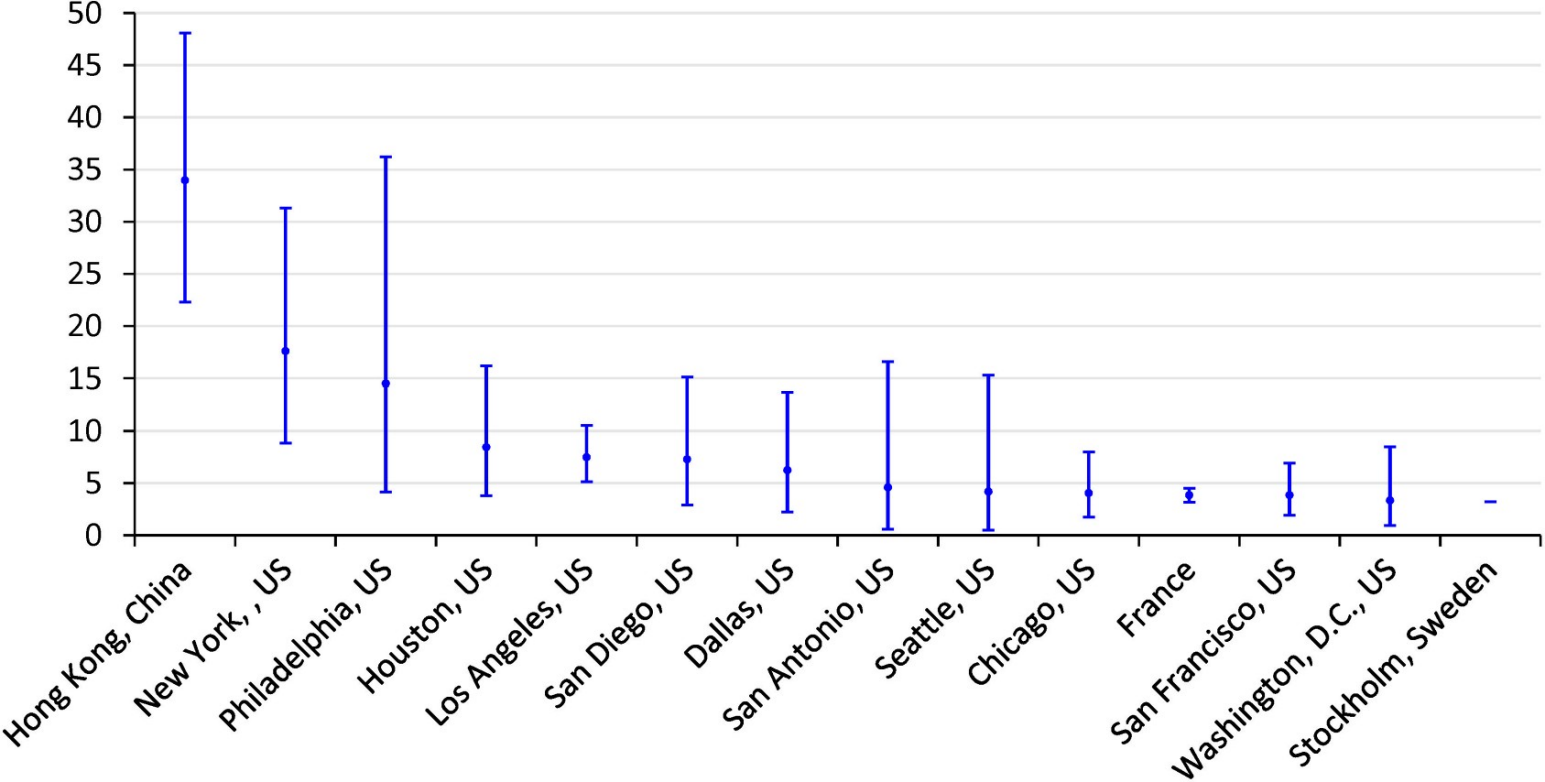
Comparison of fatal injury rate for cycling in Hong Kong with that in other regions worldwide (dots: fatality rate per billion minutes cycled; solid lines: 95% CI; Inclusion criteria: fatalities were defined as those who died immediately at the crash scene or within 30 days of the collision, and cycling time was used as the exposure; data sources: Schneider et al. [25], McAndrews [33], and Bouaoun et al. [34]).

Compared with other regions worldwide, Hong Kong was among the most dangerous areas for cycling during 2010–2012 (see Fig 3 and S3 Table). Per billion minutes cycled, cycling in Hong Kong (fatality rate: 33.96, 95% CI: 22.29 to 48.07) was associated with a higher rate of fatal injury than in Stockholm, Sweden (fatality rate: 3.20), France (fatality rate: 3.83, 95% CI: 3.17 to 4.50), and most US metropolitan areas, such as New York (fatality rate: 17.60, 95% CI: 8.83 to 31.30), Houston (fatality rate: 8.42, 95% CI: 3.79 to 16.20), Los Angeles (fatality rate: 7.47, 95% CI: 5.11 to 10.50), and Chicago (fatality rate: 4.03, 95% CI: 1.73 to 7.97).

## Discussion

Over the past two decades, Hong Kong has witnessed a sharp increase in the absolute number of cyclists injured in traffic crashes. The risk of crash involvement for cyclists has also increased substantially. Per minutes traveled, our results indicate that cyclists were more likely to be injured and fatally injured than pedestrians, private car drivers, private car passengers, taxi passengers, public bus passengers, and minibus passengers. A comparison of Hong Kong with other regions suggests that Hong Kong was among the most dangerous areas for cycling in terms of fatality rate per billion minutes cycled. The large number of injuries sustained by cyclists, along with the striking annual increase rate and the significantly higher risk of crash involvement, explicitly illustrates that cyclist injuries have become an appreciable public health challenge in Hong Kong.

One might plausibly attribute the sharp increase in the number of cyclist injuries to greater engagement in cycling [35]. The travel characteristics survey indeed estimated a roughly 2% growth in cycling time between 2002 and 2011 [4]. This negligible increase in bicycle use, however, is incapable of accounting for the nearly twofold increase in cyclist injuries during 2002–2011. Moreover, note that we derived our injury data directly from police reports. As injuries to cyclists are less likely to be recorded by the police than injuries to other road users [36], this increase in the number of cyclist injuries in police reports may be due to the continuous improvement in the quality of police data. However, evidence is insufficient to confirm this.

The road system in Hong Kong has long been dominated by motorists. The societal status of cycling as a marginal mode of transportation reinforces the perception of cyclists as intruders rather than an integral part of the traffic system [37]. Consequently, it seems unrealistic to expect motorists, most of whom rarely ride a bicycle and never become accustomed to driving alongside cyclists, to give more consideration to cyclists. During 2013–2017, 46 cyclists were fatally injured in traffic crashes, more than 80% of which occurred on carriageways without any physical separation [9]. Although a total of 219 km of bicycle paths have been available for use since 2014, most of them are fragmentarily located in newly developed towns in the New Territories. The lack of cycling infrastructure in Hong Kong Island and Kowloon makes cycling not only extremely unsafe but also inconvenient, unpleasant, and impractical, as commuters who use cycling as their daily mode of transportation have to share the roads with fast-moving motor vehicles. In addition to the inadequate cycling infrastructure in built-up areas, traffic education and training towards safe cycling also fall behind in Hong Kong. Unlike in many European countries, which provide mandatory traffic education on cycling skills to elementary school students [38], road safety education for primary pupils in Hong Kong focuses primarily on safe crossing for pedestrians. Additionally, cycling is not permitted in most public parks and playgrounds [6]. It is therefore not surprising that most people are poorly prepared to ride their bicycles on roads. Twenty years ago, only one out of ten cyclists were reported to wear a helmet at the time of injury [9]. Unfortunately, little has changed regarding helmet use among cyclists in Hong Kong in the past two decades, despite extensive evidence that the use of bicycle helmet significantly reduces the probability of head and facial injuries during impact [39]. The increased likelihood of crash involvement, combined with the increased likelihood of injury given a crash, contributes to the vulnerability of cyclists in Hong Kong.

Bicycle-related fatalities and injuries are not inevitable or unpreventable. In fact, it is beyond doubt that they can be lowered to acceptable levels by introducing multifaceted interventions aimed at reducing environmental hazards and removing barriers to cycling. Decades of successful experience from the Netherlands, Germany, and Denmark have demonstrated that cycling can be made a safe and attractive alternative to travel around cities [38]. Recent evidence from Boston also suggests that the expansion of bicycle infrastructure was associated with a significant reduction in cyclist injury rate and a large increase in cycling levels there [40]. It is time for Hong Kong to reconsider its safety strategies and to take action to curb the rapid increase in bicycle casualties. A range of countermeasures with proven effectiveness should be promptly implemented to improve the safety of these vulnerable road users. Such countermeasures might include improving motorists’ awareness of cyclists [41], traffic calming in residential neighborhoods [42], upgrading existing on-road bicycle lanes [43] or off-road bicycle paths [44] in the New Territories to consecutively separate cyclists from motor vehicles on heavily traveled roads, systematic training on cycling skills [45], enhancing the conspicuity of cyclists [46], and promoting helmet use among cyclists [39]. Improved safety would encourage more people to cycle on a regular basis for daily travel, accompanied by health benefits, mobility options, independence, and fun.

This study is not without limitations. Our injury data were obtained from police reports. Despite the potential under-reporting of less severe injuries [36], it is the only representative data source publicly available for such a long-time span. The same dataset is also readily used by local authorities for safety policymaking [6,8,10,29,30,47,48]. One major challenge to quantifying the danger of cycling is the lack of reliable data on exposure [49]. Similar to many jurisdictions around the world, data available to estimate the exposure are limited in Hong Kong. The use of quasi-induced exposure allows us to elaborately measure the relative risk of crash involvement for cycling annually. However, the judgment of responsibility by the police may be somewhat arbitrary [50]. The underlying at-fault assumption also needs further verification [51]. Because the travel characteristics survey is self-reported, our estimated total amount of time traveled is probably subject to the recall bias. Furthermore, due to the limited number of studies using comparable police-recorded crashes and household travel surveys to estimate the risk of cycling per minutes cycled, our comparison of the fatal injury rate in Hong Kong with that of other cities was mainly restricted to US metropolitan areas. Future research extending our analysis to a larger set of regions, especially Asian cities, is advocated.

In conclusion, traffic injuries to cyclists increased substantially over the past two decades and have become a considerable public health burden in Hong Kong. Although the health benefits of cycling broadly outweigh the harms caused by traffic injuries and air pollution [52], and cycling has been empirically proved to be statistically less dangerous than many other recreational activities in New Zealand [53], the promotion of cycling in Hong Kong, particularly in urbanized areas with limited space for provision of cycling facilities, should be based on the condition that the safety and mobility of these vulnerable road users are consistently improved. Public health experts should work together with cycling communities, urban planners, traffic engineers, architects, environmentalists, and government officials at all levels to make Hong Kong a more cyclable city.

## Supporting information

S1 Table Number of cyclists, pedestrians, motorcyclists, private car drivers, private car passengers, taxi passengers, public bus passengers, and minibus passengers injured in road traffic crashes in Hong Kong, 1998–2017. (DOCX)

S2 Table Relative risk of crash involvement for cyclists, measured by quasi-induced exposure, in Hong Kong, 1998–2017. (DOCX)

S3 Table Comparison of fatal injury rate for cycling per billion minutes in Hong Kong with that in other regions worldwide. (DOCX)
