# Supplementary material for: Cyclists injured in traffic crashes in Hong Kong: A call for action
Source: PLoS One. 2019 Aug 9;14(8):e0220785. doi: 10.1371/journal.pone.0220785 (PMC6688837; doi:10.1371/journal.pone.0220785)
Supplement: S1 Table — (DOCX) [file pone.0220785.s001.docx]

**Supporting information**

**S1 Table.** Number of cyclists, pedestrians, motorcyclists, private car drivers, private car passengers, taxi passengers, public bus passengers, and minibus passengers injured in road traffic crashes in Hong Kong, 1998–2017.

| Year | Cyclists | | Pedestrians | | Motorcyclists | | Private car  drivers | | Private car  passengers | | Taxi  passengers | | Public bus^†^  passengers | | Minibus  passengers | |
| --- | --- | --- | --- | --- | --- | --- | --- | --- | --- | --- | --- | --- | --- | --- | --- | --- |
|  | No. | % | No. | % | No. | % | No. | % | No. | % | No. | % | No. | % | No. | % |
| 1998 | 683 | 3.58 | 4932 | 25.85 | 2040 | 10.69 | 2109 | 11.06 | 2539 | 13.31 | 889 | 4.66 | 1480 | 7.48 | 728 | 3.82 |
| 1999 | 1042 | 5.37 | 4830 | 24.87 | 2209 | 11.37 | 2081 | 10.71 | 2196 | 11.31 | 833 | 4.29 | 1718 | 9.01 | 741 | 3.82 |
| 2000 | 980 | 5.04 | 4785 | 24.63 | 2440 | 12.56 | 2134 | 10.98 | 2236 | 11.51 | 922 | 4.75 | 1515 | 7.80 | 759 | 3.91 |
| 2001 | 1225 | 6.03 | 4978 | 24.50 | 2602 | 12.81 | 2137 | 10.52 | 2115 | 10.41 | 933 | 4.59 | 1749 | 9.00 | 846 | 4.16 |
| 2002 | 1281 | 6.22 | 4805 | 23.33 | 2474 | 12.01 | 2294 | 11.14 | 2241 | 10.88 | 889 | 4.32 | 1685 | 8.29 | 834 | 4.05 |
| 2003 | 1408 | 7.69 | 4517 | 24.67 | 2486 | 13.58 | 1783 | 9.74 | 1594 | 8.71 | 790 | 4.31 | 1600 | 7.77 | 699 | 3.82 |
| 2004 | 1627 | 8.39 | 4577 | 23.59 | 2518 | 12.98 | 1777 | 9.16 | 1593 | 8.21 | 866 | 4.46 | 1580 | 8.63 | 919 | 4.74 |
| 2005 | 1407 | 7.23 | 4404 | 22.91 | 2580 | 13.42 | 1843 | 9.59 | 1616 | 8.41 | 966 | 5.03 | 1649 | 8.50 | 753 | 3.92 |
| 2006 | 1354 | 7.17 | 4233 | 22.43 | 2568 | 13.61 | 1858 | 9.84 | 1608 | 8.52 | 929 | 4.92 | 1676 | 8.72 | 741 | 3.93 |
| 2007 | 1398 | 7.12 | 4078 | 20.78 | 2727 | 13.90 | 2006 | 10.22 | 1463 | 7.45 | 1024 | 5.22 | 1837 | 9.73 | 868 | 4.42 |
| 2008 | 1439 | 7.70 | 3823 | 20.46 | 2388 | 12.78 | 1778 | 9.52 | 1416 | 7.58 | 968 | 5.18 | 1710 | 8.71 | 724 | 3.87 |
| 2009 | 1581 | 8.72 | 3583 | 19.75 | 2368 | 13.06 | 1806 | 9.96 | 1498 | 8.26 | 934 | 5.15 | 1664 | 8.91 | 753 | 4.15 |
| 2010 | 1768 | 9.24 | 3898 | 20.38 | 2224 | 11.63 | 1791 | 9.37 | 1489 | 7.79 | 1035 | 5.41 | 1818 | 10.02 | 846 | 4.42 |
| 2011 | 2194 | 11.08 | 3840 | 19.39 | 2146 | 10.84 | 1947 | 9.83 | 1416 | 7.15 | 1104 | 5.57 | 1841 | 9.63 | 862 | 4.35 |
| 2012 | 2442 | 12.08 | 3822 | 18.91 | 2072 | 10.25 | 2026 | 10.02 | 1438 | 7.12 | 1190 | 5.89 | 1946 | 9.83 | 846 | 4.19 |
| 2013 | 2413 | 11.72 | 3694 | 17.94 | 2041 | 9.91 | 2044 | 9.92 | 1609 | 7.81 | 1317 | 6.39 | 2129 | 10.53 | 847 | 4.11 |
| 2014 | 2315 | 11.66 | 3604 | 18.15 | 2113 | 10.64 | 2080 | 10.48 | 1496 | 7.54 | 1195 | 6.02 | 1975 | 9.59 | 772 | 3.89 |
| 2015 | 2395 | 11.75 | 3513 | 17.24 | 2136 | 10.48 | 2311 | 11.34 | 1531 | 7.51 | 1204 | 5.91 | 1936 | 9.75 | 861 | 4.22 |
| 2016 | 1978 | 9.83 | 3429 | 17.03 | 2177 | 10.81 | 2480 | 12.32 | 1651 | 8.20 | 1123 | 5.58 | 1895 | 9.30 | 817 | 4.06 |
| 2017 | 1779 | 8.95 | 3090 | 15.54 | 2109 | 10.60 | 2708 | 13.62 | 1770 | 8.90 | 1017 | 5.11 | 1945 | 9.66 | 745 | 3.75 |

^†^ Franchised pubic bus, including New World First Bus, Kowloon Motor Bus, China Motor Bus, New Lantau Bus, City Bus, Long Win Bus, and unconfirmed franchised bus.
