# Supplementary material for: Cyclists injured in traffic crashes in Hong Kong: A call for action
Source: PLoS One. 2019 Aug 9;14(8):e0220785. doi: 10.1371/journal.pone.0220785 (PMC6688837; doi:10.1371/journal.pone.0220785)
Supplement: S2 Table — (DOCX) [file pone.0220785.s002.docx]

**Supporting information**

**S2 Table.** Relative risk of crash involvement for cyclists, measured by quasi-induced exposure in Hong Kong, 1998–2017.

| Year | Odds ratio | 95% CI^†^ |
| --- | --- | --- |
| 1998 | 1.00 |  |
| 1999 | 1.06 | (0.86, 1.30) |
| 2000 | 0.71 | (0.58, 0.87) |
| 2001 | 0.40 | (0.33, 0.49) |
| 2002 | 1.57 | (1.28, 1.93) |
| 2003 | 2.00 | (1.62, 2.46) |
| 2004 | 2.83 | (2.29, 3.50) |
| 2005 | 2.29 | (1.85, 2.83) |
| 2006 | 2.81 | (2.25, 3.50) |
| 2007 | 2.06 | (1.68, 2.54) |
| 2008 | 1.75 | (1.43, 2.15) |
| 2009 | 2.42 | (1.97, 2.98) |
| 2010 | 1.73 | (1.42, 2.11) |
| 2011 | 1.60 | (1.32, 1.93) |
| 2012 | 1.76 | (1.46, 2.12) |
| 2013 | 1.78 | (1.48, 2.15) |
| 2014 | 2.44 | (2.01, 2.96) |
| 2015 | 2.51 | (2.06, 3.04) |
| 2016 | 2.23 | (1.83, 2.71) |
| 2017 | 2.21 | (1.82, 2.69) |

^†^ Confidence interval.
