# Supplementary material for: Cyclists injured in traffic crashes in Hong Kong: A call for action
Source: PLoS One. 2019 Aug 9;14(8):e0220785. doi: 10.1371/journal.pone.0220785 (PMC6688837; doi:10.1371/journal.pone.0220785)
Supplement: S3 Table — (DOCX) [file pone.0220785.s003.docx]

**Supporting information**

**S3 Table.** Comparison of fatal injury rate for cycling per billion minutes in Hong Kong with that in other regions worldwide.

| Areas | Data source^†^ | Period | Rate^‡^ | 95% CI^*^ |
| --- | --- | --- | --- | --- |
| Hong Kong, China |  | 2010–2012 | 33.96 | (22.29, 48.07) |
| Stockholm, Sweden | McAndrews [33] | 1999–2007 | 3.20 |  |
| France | Bouaoun et al. [34] | 2007–2008 | 3.83 | (3.17, 4.50) |
| Chicago, United States | Schneider et al. [25] | 2007–2011 | 4.03 | (1.73, 7.97) |
| Dallas, United States |  |  | 6.22 | (2.24, 13.70) |
| Houston, United States |  |  | 8.42 | (3.79, 16.20) |
| Los Angeles, United States |  |  | 7.47 | (5.11, 10.50) |
| New York, United States |  |  | 17.60 | (8.83, 31.30) |
| Philadelphia, United States |  |  | 14.50 | (4.12, 36.20) |
| San Antonio, United States |  |  | 4.57 | (0.55, 16.60) |
| San Diego, United States |  |  | 7.27 | (2.90, 15.10) |
| San Francisco, United States |  |  | 3.83 | (1.89, 6.92) |
| Seattle, United States |  |  | 4.18 | (0.49, 15.30) |
| Washington, D.C., United States |  |  | 3.33 | (0.92, 8.47) |

^†^ Inclusion criteria: fatalities were defined as those who died immediately at the crash scene or within 30 days of the collision, and cycling time was used as the exposure.

^‡^ Fatal injury rate per billion minutes.

^*^ Confidence interval.
